# Gestational Age-Dependent Associations Between Mycoplasma/Ureaplasma Colonization and Inflammatory Placental Lesions in Preterm Birth

**DOI:** 10.3390/jcm15103868

**Published:** 2026-05-18

**Authors:** Bilge Çetinkaya Demir, Sofia Zadran, Nazmiye Ülkü Tüzemen, Selva Kabul, Hilal Özkan, Cüneyt Özakın

**Affiliations:** 1 Department of Perinatology, Uludag University Faculty of Medicine, Bursa Uludag University, 16059 Bursa, Turkey; 2 Department of Obstetrics and Gynecology, Uludag University Faculty of Medicine, Bursa Uludag University, 16059 Bursa, Turkey; sofiamirzaali@gmail.com; 3 Department of Medical Microbiology, Uludag University Faculty of Medicine, Bursa Uludag University, 16059 Bursa, Turkey; utuzemen@uludag.edu.tr (N.Ü.T.);; 4 Department of Pathology, Uludag University Faculty of Medicine, Bursa Uludag University, 16059 Bursa, Turkey; selva@uludag.edu.tr; 5 Department of Neonatology, Uludag University Faculty of Medicine, Bursa Uludag University, 16059 Bursa, Turkey; hilalozkan@uludag.edu.tr

**Keywords:** preterm birth, *Mycoplasma hominis*, *Ureaplasma urealyticum*, placental histopathological chorioamnionitis

## Abstract

**Background:** Infection and inflammation are key contributors to spontaneous preterm birth (PTB), but the relationship between genitourinary microbial colonization and placental inflammatory pathology across preterm subgroups remains unclear. **Methods:** In this case–control study, women with PTB were compared with gestational age-matched controls. Urine cultures, Mycoplasma/Ureaplasma screening, inflammatory markers, and placental histopathology were analyzed. Early (24–33 weeks) and late (34–36 weeks) preterm births were evaluated separately. **Results:** Clinical risk factors were more common in PTB cases (87.0% vs. 68.7%, *p* = 0.001), particularly PPROM and fetal growth restriction. Conventional urine culture positivity did not differ between groups. Mycoplasma/Ureaplasma colonization was more frequent in controls (41.2% vs. 15.4%, *p* < 0.001). Early PTB was strongly associated with placental inflammation, including higher rates of histological chorioamnionitis, composite inflammatory lesions, placental culture positivity, and elevated CRP compared with late PTB. **Conclusions:** Early PTB may represent a distinct infection-associated phenotype characterized by prominent placental inflammation, whereas late PTB demonstrates a weaker inflammatory profile.

## 1. Introduction

Preterm birth (PTB) remains the leading cause of neonatal mortality and a major contributor to long-term neurodevelopmental impairment worldwide [1]. Despite advances in obstetric and neonatal care, approximately 15 million infants are born preterm each year, accounting for more than one million deaths annually. Survivors face increased risks of respiratory morbidity, sepsis, cerebral palsy, and lifelong neurocognitive and behavioral impairments [2,3]. The persistent global burden of PTB underscores the need to better elucidate its underlying mechanisms and identify modifiable risk factors.

Parturition, whether at term or preterm, is driven by a coordinated biological cascade involving enhanced myometrial contractility, cervical remodeling, and rupture of the fetal membranes [4,5]. This transition is mediated by a shift from anti-inflammatory to pro-inflammatory signaling within gestational tissues, characterized by activation of cytokines (e.g., IL-1, IL-6), chemokines (e.g., IL-8), and contraction-associated proteins, including prostaglandins and oxytocin receptors [4,6]. In preterm labor, this inflammatory cascade is often prematurely activated [4].

PTB is increasingly conceptualized as a multifactorial syndrome rather than a single disease entity [4,5,7,8]. Converging pathways—including infection and inflammation, uteroplacental ischemia, decidual hemorrhage, uterine overdistension, maternal stress, and immune maladaptation—can independently or synergistically precipitate early parturition [5,6,7,8]. Notably, spontaneous PTB frequently occurs in women without identifiable clinical risk factors, and in more than half of cases the initiating trigger remains undefined [6,7].

Among the proposed mechanisms, infection-driven inflammation represents one of the most consistently implicated pathways [9]. Microbial invasion of the amniotic cavity is identified in up to 30–40% of preterm births, particularly in cases associated with preterm premature rupture of membranes (PPROMs) [9]. Ascending infection from the lower genital tract constitutes the predominant route, enabling vaginal microorganisms to access the chorioamniotic membranes and amniotic fluid [10]. The resulting host immune response promotes cytokine release, prostaglandin synthesis, membrane degradation, and uterine activation. Importantly, intrauterine infection may be clinically silent, with histological chorioamnionitis and funisitis often representing the only evidence of a fetal inflammatory response [11].

*Mycoplasma hominis* and *Ureaplasma urealyticum* are among the most frequently isolated microorganisms in the lower genital tract and are uniquely adapted to mucosal colonization due to their lack of a cell wall and intrinsic resistance to β-lactam antibiotics [12,13,14]. These organisms have been repeatedly detected in amniotic fluid and intrauterine tissues in cases of PTB and PPROM [13,15,16]. Experimental and clinical studies suggest that they can induce robust inflammatory responses within gestational tissues, potentially acting as key mediators of preterm parturition [15,16]. However, their pathogenic role remains controversial, as colonization does not invariably translate into adverse pregnancy outcomes [16].

A critical limitation of the existing literature is the predominant focus on amniotic fluid cultures, with comparatively limited data integrating placental microbiological findings with detailed histopathological evaluation [16]. Given that the placenta represents both a target and mediator of inflammatory injury, simultaneous assessment of microbial presence and placental inflammatory response may provide more direct insight into infection-associated PTB mechanisms.

The primary aim of our study was to evaluate conventional microbiological findings and their association with preterm birth within the scope of routinely available clinical diagnostics. Specifically, we aimed to determine the prevalence of *M. hominis* and *U. urealyticum* in placental cultures obtained from preterm births and to assess their association with placental histopathological inflammation and adverse pregnancy outcomes. By integrating microbiological and histopathological data, we sought to better elucidate the potential contribution of these organisms to the inflammatory cascade underlying preterm parturition.

## 2. Materials and Methods

This prospective, single-center study was conducted at a tertiary university hospital between March 2023 and March 2024. Ethical approval was obtained from the Institutional Review Board of the Faculty of Medicine (approval no: 2023-5/18). Written informed consent was obtained from all participants prior to enrollment. The study was conducted in accordance with the principles of the Declaration of Helsinki.

Pregnant women aged 18–45 years with a gestational age between 24^0^ and 36^6^ weeks were eligible for inclusion. The preterm birth (PTB) group consisted of women who delivered preterm due to:

1. Spontaneous preterm labor with intact membranes,
2. Preterm premature rupture of membranes (PPROM), or
3. Medically indicated (iatrogenic) preterm birth.

The control group included pregnant women within the same gestational age range who attended routine prenatal care and had no signs or symptoms of active labor.

Preterm births were categorized according to gestational age as:

- Extremely preterm: 24^0^–27^6^ weeks
- Very preterm: 28^0^–31^6^ weeks
- Moderate preterm: 32^0^–33^6^ weeks
- Late preterm: 34^0^–36^6^ weeks

Established risk factors for PTB were recorded and included: prior preterm birth, PPROM in the index pregnancy, conception via in vitro fertilization, multiple gestation, cervical insufficiency, maternal obesity (BMI ≥ 30 kg/m^2^), polyhydramnios, fetal growth restriction (FGR), and maternal smoking. FGR was defined as an estimated fetal weight (EFW) or abdominal circumference (AC) below the 10th percentile for gestational age according to standardized reference charts.

In cases of preterm delivery, placental tissue samples (1 × 1 × 1 cm) were collected from the maternal surface under sterile conditions and submitted to the Department of Medical Microbiology, Bacteriology Laboratory for *Mycoplasma hominis* and *Ureaplasma urealyticum* culture analysis. *M. hominis* and *U. urealyticum* were identified using the Mycoplasma IES kit (Autobio Diagnostics, National Eco&Tech Zone, Zhengzhou, China), which detects ammonia production via urease (for *U. urealyticum*) or arginase (for *M. hominis*), indicated by a pH-dependent colorimetric change. Placental specimens were subjected to both *M. hominis* and *U. urealyticum* culture and histopathological examination to evaluate inflammatory responses and structural abnormalities.

The Mycoplasma IES kit comprises lyophilized powder and a separate diluent designated for the medium. Under aseptic conditions, the surgically excised placental specimen was placed in the diluent and sent to the Bacteriology Laboratory in a diluent tube. Subsequently, all of the diluent comprising the surgical material was combined with the powder tube, and the procedure was executed in accordance with the manufacturer’s guidelines.

The kit comprises 30 wells, including growth-control wells, species-specific identification wells, and antibiotic susceptibility wells. A color change from yellow to red was interpreted as microbial growth, and a bacterial load ≥10^4^ CFU/mL was considered clinically significant. The antibiotics included in the kit are pristinamycin, minocycline, josamycin, erythromycin, roxithromycin, clindamycin, ofloxacin, ciprofloxacin, clarithromycin, tetracycline, and levofloxacin. It is important to note that a single concentration well is designated solely for pristinamycin, while the remaining antibiotics feature two concentration wells each.

Furthermore, concurrent midstream urine samples were collected from pregnant women for microbiological culture, as well as for the specific cultures of *M. hominis* and *U. urealyticum.* For patients with preterm birth, concordance between placental and urine cultures for *M. hominis* and *U. urealyticum* was investigated. Urine samples were cultured on 5% sheep blood agar and Eosin Methylene Blue (EMB) agar plates (BD Diagnostic Systems, Franklin Lakes, NJ 07417, USA), followed by incubation at 37 °C for one night. Identification of the samples was performed using matrix-assisted laser desorption ionization time-of-flight mass spectrometry (MALDI-TOF MS) (Bruker Daltonik, Bremen, Germany) in conjunction with the IVD version 12.0 database. Antibiotic susceptibility testing was conducted using the BD Phoenix^TM^ M50 Automated System (SKU/REF 443624) (BD Diagnostic Systems, Franklin Lakes, NJ 07417, USA ) in accordance with the guidelines established by the European Committee on Antimicrobial Susceptibility Testing (EUCAST). Additionally, for the culture of *M. hominis* and *U. urealyticum*, the Mycoplasma IES kit was utilized in accordance with the manufacturer’s instructions.

In addition, urine samples and vaginal swabs were collected from both study and control participants under sterile conditions. Urine samples were analyzed at the Biochemistry Laboratory for pyuria, defined as ≥10 leukocytes/mm^3^ in uncentrifuged urine. Vaginal swabs were similarly cultured at the Department of Medical Microbiology, Bacteriology Laboratory for the detection of potential pathogens. Vaginal samples were cultured on 5% sheep blood agar and chocolate agar plates and incubated at 37 °C for a duration of two nights. The identification of the samples was conducted utilizing MALDI-TOF mass spectrometry. Antibiotic susceptibility testing was performed using the BD Phoenix M50 System, in accordance with the guidelines established by the EUCAST.

No *Lactobacillus* species were detected in the study samples. Since the specimens were processed in the bacteriology laboratory, no selective or differential mycological media were used. *Candida albicans* isolates were detected through growth on blood agar plates.

Placental sampling and histopathological examination were performed in accordance with the recommendations of the Amsterdam Placental Workshop Group consensus statement [17], with minor modifications. Following macroscopic examination, including documentation of placental weight and gross features, standardized tissue sampling was carried out. Extraplacental membranes were sampled using the roll technique. The umbilical cord was sampled in three separate segments. At least four full-thickness sections of the placental parenchyma were obtained, ensuring inclusion of the chorionic plate. These sections were taken from representative areas of the placental disc. In cases with grossly identified lesions, additional targeted sections were obtained. All specimens were fixed in 10% neutral buffered formalin, routinely processed, and embedded in paraffin. Sections of 3–4 µm thickness were prepared and stained with hematoxylin and eosin (H&E) for histopathological evaluation. Inflammatory lesions were evaluated and classified according to the Amsterdam Placental Workshop Group consensus criteria [17]. Acute chorioamnionitis (maternal inflammatory response) was staged based on the anatomical extent of inflammation (stage 1: subchorionitis; stage 2: involvement of the chorion and amnion; stage 3: necrotizing chorioamnionitis) and graded according to severity (grade 1: mild to moderate; grade 2: severe, characterized by confluent neutrophilic infiltrates or microabscess formation). The fetal inflammatory response (funisitis) was similarly staged (stage 1: chorionic vasculitis or umbilical phlebitis; stage 2: involvement of the umbilical arteries; stage 3: necrotizing funisitis) and graded as mild (grade 1) or severe (grade 2). Cases with abscess formation were considered indicative of a severe inflammatory response (grade 2). The extent of abscess formation was recorded based on its distribution and anatomical involvement (focal, multifocal, or diffuse; and location including membranes, placental parenchyma, or umbilical cord, when applicable).

A composite inflammatory placental lesion variable was generated to enhance analytical power and reflect infection-related pathology. This variable was defined as the presence of at least one of the following histopathological findings: chorioamnionitis, funisitis, placental abscess, or necrosis. Secondary analyses evaluated the association between placental culture positivity and this composite inflammatory outcome.

Comparisons between early (24–33 weeks) and late (34–36 weeks) preterm groups were performed to assess the association between microbial colonization and inflammatory placental lesions. Logistic regression analysis was conducted to evaluate independent predictors of early preterm birth.

Demographic characteristics, obstetric history, and predefined PTB risk factors were recorded. Laboratory markers of systemic inflammation, including C-reactive protein (CRP) levels and white blood cell (WBC) counts, were measured at admission. Neonatal outcomes included birth weight and Apgar scores at 1 and 5 min.

### Statistical Analysis

Statistical analyses were performed using SPSS version 26.0 (IBM Corp., Armonk, NY, USA). Continuous variables were assessed for normality using the Kolmogorov–Smirnov test. Normally distributed variables were expressed as mean ± standard deviation (SD), while non-normally distributed variables were presented as median (minimum–maximum). Categorical variables were expressed as counts and percentages. Comparisons between groups were performed using Pearson’s Chi-square or Fisher’s exact test for categorical variables and the Mann–Whitney U test for non-parametric continuous variables, as appropriate. A two-tailed *p*-value < 0.05 was considered statistically significant. A priori sample size calculation was performed using G-Power version 3.1. Assuming a prevalence of placental *U. urealyticum* of 30% in the PTB group and 10% in controls, with a two-sided α level of 0.05 and 80% power, a minimum of 90 participants per group was required to detect a statistically significant difference.

Given the observed prevalence of inflammatory placental lesions (approximately 20% in early preterm vs. 3% in late preterm births), the available sample size (*n* = 117 preterm deliveries) provided adequate power (>80%) to detect moderate-to-large effect sizes (OR ≥ 5) between gestational age groups. However, due to the low prevalence of placental culture positivity (~5%), analyses involving microbial culture alone may be underpowered to detect small-to-moderate associations.

## 3. Results

During the study period, 1050 deliveries were recorded at our institution. Of these, 166 women declined participation. Among the remaining eligible population, 117 women (13.2%) presenting with clinical features of preterm labor were enrolled in the preterm birth (PTB) group, while 99 asymptomatic pregnant women at comparable gestational ages constituted the control group.

The mean maternal age in the PTB group was 29.29 ± 4.75 years (median 29; IQR 19–43), and did not differ significantly from that of the control group (*p* > 0.05).

Obstetric characteristics differed between groups. Gravidity (*p* = 0.012), parity (*p* = 0.009), and number of living children (*p* = 0.007) were significantly lower in the PTB group compared with controls. In contrast, no significant differences were observed between groups in terms of maternal age, history of abortion, or daily cigarette consumption (all *p* > 0.05) (Table 1).

In the PTB group, the mean gestational age at delivery was 33.41 ± 3.10 weeks. In the PTB group median birth weight was 2190 g (1300–3100 g). Median APGAR scores in 1st minute was 7 and at 5th minute was 8. Deliveries occurred in the extremely preterm period (24–27 weeks) in 6.8% of cases (*n*:8), while 62.4% (*n*:73) occurred in the late preterm period (34–36 weeks). Cesarean delivery was performed in 76.1% of PTB cases, whereas 23.9% delivered vaginally.

At least one established risk factor for PTB was identified in 87.2% of women in the PTB group compared with 68.7% in the control group (*p* = 0.001) (Table 2). The most frequent risk factors in the PTB group were presence of preterm premature rupture of membranes (PPROM, 16.2%), fetal growth restriction (FGR, 14.5%), multiple gestation (13.7%), and smoking (10.3%). In contrast, smoking (17.2%) and in vitro fertilization (IVF) pregnancy (7.1%) were the leading risk factors in the control group. PPROM and FGR were significantly more common in the PTB group than in controls (*p* < 0.001 and *p* = 0.001, respectively), whereas IVF pregnancy, prior preterm birth, and smoking did not differ significantly between groups (*p* > 0.05).

### 3.1. Urinary Findings and Conventional Microbiology

At the time of urine sampling, the mean gestational age was 33.41 ± 3.10 weeks in the PTB group and 29.04 ± 4.13 weeks in the control group. Pyuria was detected in 18.8% of PTB patients and 11.7% of controls, while positive routine urine cultures were identified in 6.8% of cases, these differences were not statistically significant (*p* > 0.05).

The most common urinary isolates in the PTB group were *Escherichia coli* and *Klebsiella pneumoniae*. In the control group, again *E. coli* (60.0%) is the predominant microorganism (Table 3).

#### Mycoplasma and Ureaplasma Detection

Mycoplasma/Ureaplasma growth was detected in 15.4% of the PTB group and 41.2% of controls (*p* < 0.001). *Ureaplasma urealyticum* was identified in all positive cases in both groups. Additionally, *Mycoplasma hominis* was isolated in 5.0% of control patients. High colony counts (≥10^4^ CFU/mL) were observed in 77.8% of positive PTB cases and 57.5% of positive controls, without a statistically significant difference (*p* > 0.05) (Table 3).

Vaginal culture growth was identified in 10.5% of PTB patients. Pathogenic microorganisms such as *Neisseria gonorrhoeae* were not detected in any of the pregnant women, and fungal colonization was detected in only 2 patients. (*Candida albicans*, 16.7%).

Since the Mycoplasma IES system is primarily intended for detection of *M. hominis* and *Ureaplasma* spp., other Mollicutes such as *Mycoplasma genitalium* may not have been detected. Molecular confirmation methods were not performed in the present study.

### 3.2. Early vs. Late Preterm Birth: Infection–Inflammation Gradient

When preterm births were stratified by gestational age (early: 24–33 weeks; late: 34–36 weeks), a distinct gradient emerged. The mean CRP level was significantly higher in the early preterm group compared with the late preterm group (*p* = 0.045), whereas WBC count and daily cigarette consumption did not differ significantly (*p* > 0.05) (Table 4).

Although rates of urinary, vaginal, and placental microbial detection were numerically higher in the early preterm group, these differences did not reach statistical significance (*p* > 0.05). However, placental histopathological abnormalities were significantly more frequent in early preterm births (*p* = 0.004). Only three neonates in the early preterm group with placental chorioamnionitis and abscess formation died. The remaining neonates with composite inflammatory placental lesions received appropriate antibiotic treatment and were discharged home. Only one neonate in this group developed grade 3 intraventricular hemorrhage.

**Figure 1:**
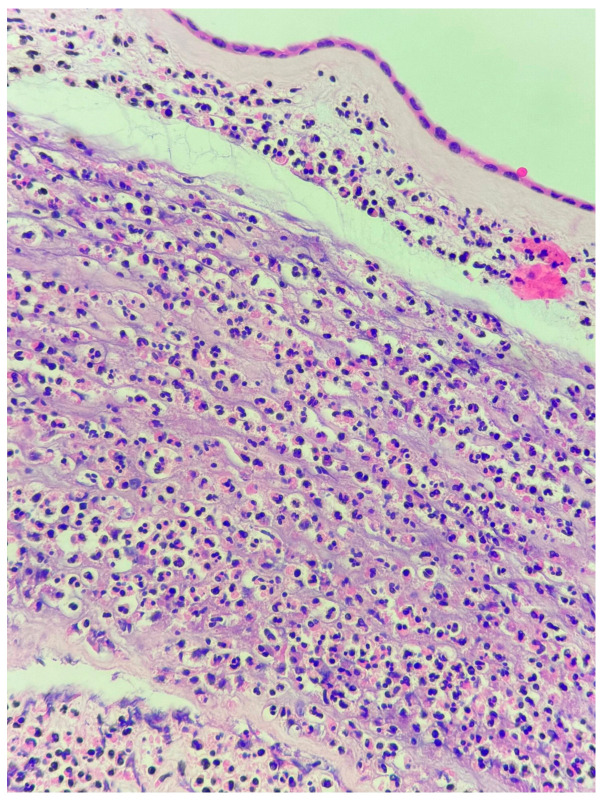
Acute chorioamnionitis. Neutrophilic infiltration involving the chorion and amnion, consistent with maternal inflammatory response (hematoxylin and eosin, original magnification ×200).

**Figure 2:**
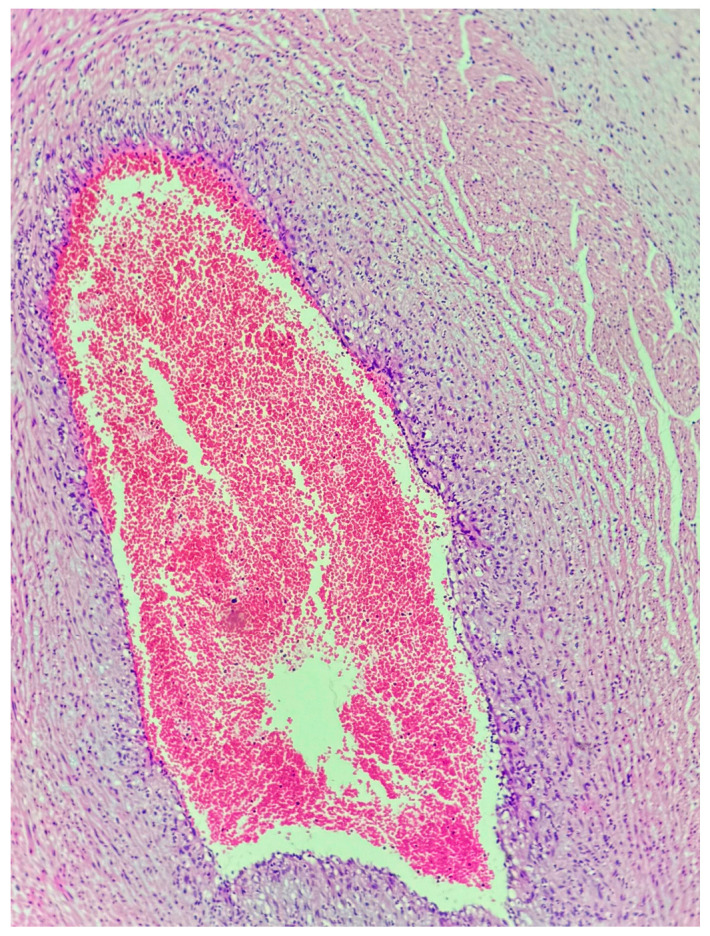
Funisitis. Neutrophilic infiltration within the umbilical cord vessel wall, representing fetal inflammatory response (hematoxylin and eosin, original magnification ×100).

**Figure 3:**
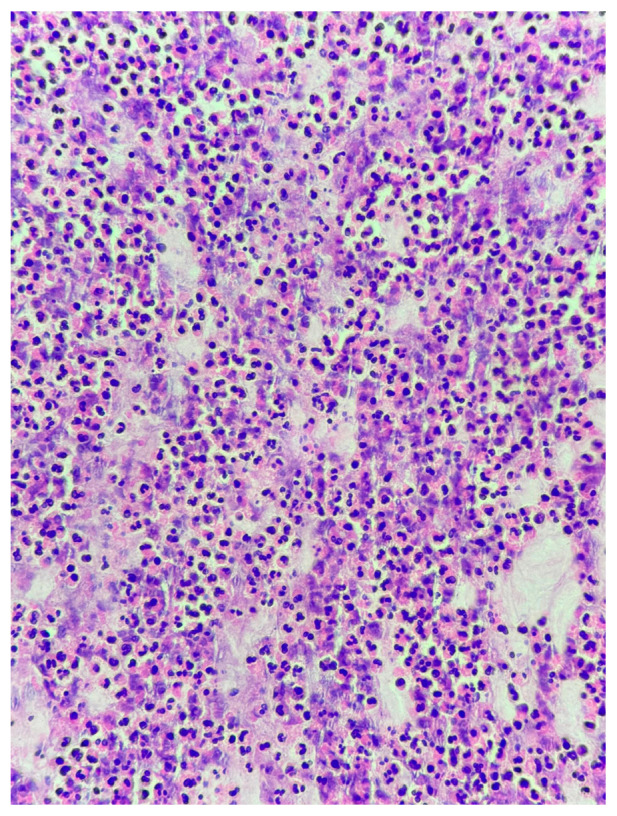
Placental abscess. Focal intraparenchymal collection of neutrophils forming an abscess within the placental parenchyma (hematoxylin and eosin, original magnification ×200).

Histological chorioamnionitis was observed in 20.5% of early preterm cases compared with 2.7% of late preterm cases (OR 9.1, *p* = 0.002). Most cases were classified as stage 2 (involvement of the chorion and amnion), with both grade 1 (mild to moderate) and grade 2 (severe) inflammatory activity observed (Figure 1). No placental abscesses were identified in the late preterm group. Funisitis did not show a statistically significant association with gestational age (*p* > 0.05). All 5 patients with placental funisitis had histological chorioamnionitis at the same time. The majority were stage 1 (chorionic vasculitis or umbilical phlebitis), while a smaller subset demonstrated stage 2 involvement (extension to the umbilical arteries). Both grade 1 and grade 2 inflammatory responses were noted (Figure 2). And all those patients presented with a diagnosis of PPROM and with high CRP levels (>15 mg/dL). All 6 patients with placental abscess had CRP levels >25 mg/dL but only one presented with PPROM. Abscess formation was identified in 6 cases, all of which were focal and located within the placental parenchyma. These findings were interpreted as indicative of a severe inflammatory response (grade 2). In four of these cases, abscess formation was accompanied by placental infarction (Figure 3). All late preterm patients (*n*:2) with a diagnosis of chorioamnionitis presented with PPROM and 5 of 9 nine patients in early preterm presented with PPROM. Only 5 of 11 patients had placental and urine culture positivity. Placental culture positivity was more frequent in early preterm deliveries (9.1%) than in late preterm deliveries (2.7%), corresponding to an odds ratio of 3.55. When a composite inflammatory placental lesion variable (chorioamnionitis, abscess, funisitis, or necrosis) was analyzed, early preterm birth was associated with a markedly increased risk compared with late preterm birth (estimated OR 8–10).

### 3.3. Summary of Key Finding

A clear gestational age-dependent gradient was observed, with higher rates of inflammatory placental pathology—and numerically higher microbial detection—at earlier gestational ages. These findings support a pathogen-driven inflammatory mechanism contributing preferentially to more severe (earlier) forms of preterm birth.

## 4. Discussion

Preterm birth (PTB) remains a leading cause of neonatal morbidity and mortality, and infection-related inflammation plays a central role in its pathogenesis, particularly at earlier gestational ages. In the present study, early preterm births (<34 weeks) were characterized by significantly higher rates of histologic chorioamnionitis and elevated maternal CRP levels compared with late preterm births. These findings support the concept that early PTB represents a distinct inflammatory phenotype and reinforce the importance of inflammatory pathways in the mechanisms leading to early delivery.

Genital mycoplasmas, particularly *Ureaplasma urealyticum*, *Ureaplasma parvum*, and *Mycoplasma hominis*, are among the microorganisms most frequently isolated from amniotic fluid and placental tissues in cases of histologic and clinical chorioamnionitis and spontaneous PTL and PROM [13,18]. The prevalence of chorioamnionitis is strongly dependent on gestational age at delivery, occurring in approximately 3–5% of placentas delivered at term but in up to 94% of placentas delivered between 21 and 24 weeks of gestation [19]. Similarly, colonization rates decline with advancing gestational age, decreasing from 79% at 23 weeks to 43% at 27 weeks [20]. Hillier et al. also demonstrated that placental evidence of infection is most common at the lowest gestational ages [21]. These observations support the concept that infection-driven mechanisms predominate in early preterm birth, whereas non-infectious etiologies such as preeclampsia or fetal growth restriction are more frequently associated with later preterm deliveries.

Consistent with this model, microbial colonization is markedly more common in spontaneous preterm deliveries than in indicated preterm births, with reported rates of 34.7% versus 3.2% [19]. Similarly, placentas delivered by cesarean delivery for preeclampsia demonstrate substantially lower bacterial recovery rates (approximately 25%) [20]. Together, these findings highlight the role of ascending infection as a major contributor to spontaneous early PTB.

Ureaplasma colonization has been associated with characteristic inflammatory patterns within placental tissues, including neutrophilic infiltration of the amnion and subchorionic regions as well as funisitis [22]. These inflammatory processes have also been linked to important neonatal morbidities, including bronchopulmonary dysplasia (pooled OR 2.39) [23], intraventricular hemorrhage [24], and neonatal systemic inflammatory response syndrome [19]. However, microbial invasion may not always be detected by conventional culture methods, particularly when infection is localized to the decidua or chorioamniotic interface or when prior antibiotic exposure reduces bacterial load. In this context, inflammatory activation rather than microbial detection alone may be a more relevant determinant of early delivery [25,26].

An interesting observation in our cohort was the higher rate of *Mycoplasma*/*Ureaplasma* colonization detected in control urine samples compared with the PTB group. This finding underscores the distinction between lower genital tract colonization and clinically significant intrauterine infection. Detection of microorganisms directly in intrauterine compartments such as the placenta or amniotic fluid has been shown to yield stronger associations with PTB than cervical or vaginal sampling alone [27,28]. Indeed, while vaginal colonization with *Ureaplasma* species is relatively common and does not reliably predict preterm labor, the presence of these organisms in amniotic fluid or placental tissues is associated with a substantially increased risk of PTB [29].

Evidence from meta-analyses further supports this association. Noda-Nicolau et al. demonstrated significant relationships between *M. hominis* and both preterm birth (OR 2.25) and PPROM (OR 2.09) [27]. In addition, Allen-Daniels et al. identified specific virulence genes in placental and amniotic fluid isolates of *M. hominis* that were associated with increased bacterial burden and preterm delivery risk [30], suggesting that strain-level variation may influence pathogenic potential. Studies focusing on extremely preterm deliveries (<28 weeks) report microorganism recovery rates ranging from 43% to 79%, with decreasing prevalence as gestational age advances [20]. In contrast, investigations of late preterm placentas (32–36 weeks) report substantially lower *Ureaplasma* detection rates of 5.5–10.6% [13]. Differences between spontaneous and indicated preterm deliveries are similarly pronounced, with colonization rates of 34.7% versus 3.2% [22] and 43.9% versus 2.7% reported in separate studies [31].

Importantly, microbial colonization alone may be insufficient to trigger preterm labor in the absence of a measurable inflammatory response, suggesting that host inflammatory activation may represent the key mediator linking microbial exposure to adverse perinatal outcomes [19,31]. Studies reporting the strongest associations between genital mycoplasmas and PTB have typically relied on direct placental or fetal compartment sampling [19,22] and have focused on populations with spontaneous preterm labor or PPROM [31]. In contrast, studies relying solely on cervical or vaginal sampling often report weaker associations [32]. Consistent with this observation, the meta-analysis by Noda-Nicolau et al. demonstrated that detection of genital mycoplasmas in fetal tissues (amniotic fluid, fetal membranes, or placenta) is associated with significantly increased odds of PTB, whereas detection confined to the cervix or vagina shows weaker associations [27].

The significantly higher CRP levels observed in early preterm births in our cohort further support the presence of a systemic inflammatory component in this phenotype. Because earlier gestational age at delivery is strongly associated with increased neonatal morbidity—including respiratory complications, infection risk, and long-term neurodevelopmental impairment—identifying markers that reflect active inflammatory processes may have important prognostic implications [33,34]. Integration of maternal inflammatory markers with placental histopathology may therefore improve risk stratification in pregnancies at risk for early PTB.

Although *Lactobacillus*-dominant vaginal microbiota is generally considered protective in pregnancy, no *Lactobacillus* species were isolated in our study samples. As the present study specifically focused on *Mollicutes*-associated microorganisms, comprehensive microbiota analysis was not performed. Future microbiome-based studies may help clarify the broader microbial dynamics associated with preterm birth.

From a clinical standpoint, these findings raise the important question of whether the detection of genital mycoplasmas should prompt treatment. No single optimal treatment protocol for Mycoplasma and Ureaplasma colonization in pregnancy has been established. Given the high prevalence of lower genital tract colonization and its limited predictive value for preterm birth, routine screening and antibiotic treatment of asymptomatic vaginal or cervical colonization cannot be universally recommended. This is supported by evidence from the largest placebo-controlled randomized trial showing no benefit of oral erythromycin, as well as multiple retrospective cohort studies in high-risk populations demonstrating no improvement in gestational age at delivery despite treatment [35].

A major challenge in management is the high treatment failure rate for Ureaplasma, reported to be as high as 77–93% with standard oral regimens, compared to lower recurrence rates for Mycoplasma [36]. While meta-analytic data suggest a modest class effect of macrolides (OR 0.72 for preterm birth) and clindamycin (OR 0.68), particularly when administered in the second trimester to high-risk women, these findings are derived from heterogeneous populations and have not been consistently replicated in large, well-designed trials [37].

In contrast, the presence of intrauterine infection or clear evidence of inflammatory activation may justify a more targeted therapeutic approach. Therefore, clinical management should be individualized, taking into account gestational age, clinical presentation, and markers of inflammation. Further prospective studies are needed to better define which patients may benefit from antimicrobial therapy and to establish optimal treatment strategies.

Interestingly, microbial culture positivity was not associated with immediate neonatal outcomes in our cohort. This observation may reflect limited statistical power or variability in host–fetal immune responses. It also suggests that placental inflammatory pathology may be more predictive of neonatal vulnerability than microbial identification alone.

A major limitation of this study is the lack of comprehensive evaluation of the vaginal microbiota. In particular, established diagnostic approaches such as Nugent and Amsel criteria were not applied, and the composition of *Lactobacillus* species was not assessed. In addition, molecular techniques that enable the detection of fastidious microorganisms, including *Metamycoplasma hominis* and *Ureaplasma* species, were not utilized. Given that the presence and load of these organisms, as well as the overall balance of the vaginal microbiome, have been increasingly implicated in the pathophysiology of preterm birth, the absence of these data limits our ability to fully interpret the role of microbial factors in our cohort. Therefore, our findings should be considered within the context of conventional microbiological assessments. Future studies incorporating molecular-based methods and detailed microbiota profiling are needed to provide a more comprehensive understanding of the relationship between vaginal microorganisms and preterm birth.

Strengths of this study include its prospective design, the simultaneous assessment of urine, vaginal, and placental cultures, and the integration of microbiological findings with histopathologic and inflammatory marker data. However, several limitations should be acknowledged. The modest sample size may have limited statistical power, and reliance on culture-based diagnostics may have underestimated microbial prevalence, particularly for fastidious organisms. The absence of molecular diagnostic techniques such as polymerase chain reaction represents an additional limitation.

Taken together, our findings support a gestational age-dependent model of PTB in which early preterm birth is more strongly associated with inflammatory placental pathology than late preterm birth. The observed discordance between microbial detection and inflammatory findings suggests that host inflammatory response may play a central role in the pathogenesis of early PTB.

## 5. Conclusions

The identification of a gestational age-dependent inflammatory phenotype in early preterm birth may have important implications for perinatal management. Our findings suggest that maternal inflammatory markers and placental histopathologic assessment may provide clinically relevant information beyond microbial culture results alone. In cases of threatened early preterm delivery, elevated inflammatory markers such as CRP may help identify pregnancies at increased risk for inflammation-associated morbidity and guide closer maternal–fetal surveillance and neonatal preparedness. Recognizing early PTB as a predominantly inflammation-driven condition may also support the development of targeted therapeutic strategies aimed at modulating the maternal–fetal inflammatory response rather than focusing solely on antimicrobial treatment. Future studies incorporating molecular diagnostics, maternal–fetal immune profiling, and longitudinal neonatal follow-up are needed to identify clinically actionable biomarkers and refine risk stratification.

## Figures and Tables

**Table 1:** Descriptive Characteristics of the PTB and Control Groups.

Variables	PTB Group (*n* = 117)	Control Group (*n* = 99)	*p*-Value ^1^
Age (years)	29.29 ± 4.75 (29, 19–43)	29.60 ± 4.75 (29, 19–42)	NS
Gravida	2.26 ± 1.39 (2, 1–9)	2.65 ± 1.36 (3, 1–7)	0.012 *
Parity	0.79 ± 1.01 (1, 0–6)	1.07 ± 0.97 (1, 0–4)	0.009 **
Abortus	0.50 ± 0.82 (0, 0–4)	0.60 ± 0.93 (0, 0–4)	NS
Living Children	0.73 ± 0.92 (1, 0–5)	1.00 ± 0.88 (1, 0–3)	0.007 **
Smoking (cigarettes/day)	0.74 ± 2.99 (0, 0–20)	1.03 ± 3.29 (0, 0–20)	NS

PTB: preterm birth. Values are presented as Mean ± SD (Median, Min–Max). ^1^ Mann–Whitney U test. * *p* < 0.05, ** *p* < 0.01 indicate statistically significant differences.

**Table 2:** Comparison of Preterm Birth Risk Factors Between the PTB and Control Groups.

Variable	PTB Group (*n* = 117)	Control Group (*n* = 99)	*p*-Value ^1^
**Presence of Risk Factor**			
– Absent	15 (12.8%)	31 (31.3%)	0.001
– Present	102 (87.2%)	68 (68.7%)	
**Risk Factors ^†^** (Patient: *n* = 102, Control: *n* = 68)			
– IVF Pregnancy	7 (6.0%)	7 (7.1%)	NS
– Multiple Pregnancy	16 (13.7%)	0 (0%)	–
– History of Preterm Labor	4 (3.4%)	2 (2.0%)	NS
– Presence of PPROM	19 (16.2%)	1 (1.0%)	0.000
– IUGR	17 (14.5%)	2 (2.0%)	0.001
– Cervical Insufficiency	4 (3.4%)	0 (0%)	–
– Maternal Obesity	5 (4.3%)	0 (0%)	–
– Polyhydramnios	4 (3.4%)	0 (0%)	–
– Smoking (Regular Smoker)	12 (10.3%)	17 (17.2%)	NS

^1^ *p*-values were calculated using Pearson chi-square and Fisher’s Exact test as appropriate. ^†^ A single individual may have more than one risk factor.

**Table 3:** Comparison of Urine Culture Results Between the PTB and Control Groups.

Variable	PTB Group (*n* = 117)	Control Group (*n* = 99)	*p*-Value ^1^
Week of Urine Sample Collection ^†^	33.41 ± 3.10 (24–36)	29.04 ± 4.13 (24–36)	
Pyuria ^††^			0.158
– Present	22 (18.8%)	11 (11.7%)	
– Absent	95 (81.2%)	83 (88.3%)	
Urine Culture			0.581
– Positive	8 (6.8%)	5 (5.1%)	
– Negative	109 (93.2%)	94 (94.1%)	
Organisms Detected in Urine Culture			
– *Escherichia coli*	4 (50.0%)	3 (60.0%)	
– *Klebsiella pneumoniae*	3 (37.5%)	0 (0.0%)	
– *Staphylococcus epidermidis*	0 (0.0%)	2 (40.0%)	
– *Proteus mirabilis*	1 (12.5%)	0 (0.0%)	
Mycoplasma/Ureaplasma Growth in Urine			0.000
– Negative	99 (84.6%)	57 (58.8%)	
– Positive	18 (15.4%)	40 (41.2%)	
Mycoplasma/Ureaplasma Species	**(*n* = 18)**	**(*n* = 40)**	–
– *Ureaplasma urealyticum*	18 (100.0%)	40 (100.0%)	
– *Mycoplasma hominis*	0 (0.0%)	2 (5.0%)	
Mycoplasma/Ureaplasma Colony Count			0.137
– <10,000 CFU/mL	4 (22.2%)	17 (42.5%)	
– ≥10,000 CFU/mL	14 (77.8%)	23 (57.5%)	

^†^ Values expressed as Mean ± SD (Median, Min–Max). ^††^ Pyuria defined as ≥10 leukocytes per mm^3^ in uncentrifuged urine. ^1^ Pearson Chi-Square test.

**Table 4:** Comparison of Risk Factors, Pyuria, Urine, Vaginal and Placental Culture Results, and Pathological Findings According to Gestational Age in the Study Group.

Variables	Early Preterm (24–33 Weeks) *n* = 44	Late Preterm (34–36 Weeks) *n* = 73	*p* ^1^
Smoking ^†^	0.68 ± 3.11 (0, 0–20)	0.78 ± 2.94 (0, 0–20)	0.736
WBC ^†^	14,485.45 ± 5192.82 (13.500, 6600–27.800)	12.831.64 ± 3914.42 (12.400, 6100–24.700)	0.093
CRP (mg/dL) ^†^	16.99 ± 16.69 (11.6, 0–66.0)	11.24 ± 13.10 (8.4, 0–84.0)	0.045
Symptoms present	31 (70.5%)	59 (80.8%)	0.197
Presence of risk factors	40 (90.9%)	62 (84.9%)	0.349
Pyuria	11 (25.0%)	11 (15.1%)	0.183
Positive culture			
Urine culture	6 (13.6%)	6 (8.3%)	0.367
Mycoplasma/Ureaplasma in urine	10 (22.7%)	8 (11.0%)	0.087
Vaginal culture	8 (18.2%)	4 (5.7%)	0.057
Placental culture	4 (9.1%)	2 (2.7%)	0.196
Pathology finding present	38 (86.4%)	45 (61.6%)	0.004
Chorioamnionitis	9 (20.5%)	2 (2.7%)	0.002
Abscess	6 (13.6%)	0 (0.0%)	–
Funisitis	4 (9.1%)	1 (1.4%)	0.066

^†^ Values expressed as Mean ± SD (Median, Min–Max). ^1^ *p*-values were calculated using Mann–Whitney U test, Pearson chi-square and Fisher’s Exact test as appropriate.

## Data Availability

The original contributions presented in this study are included in the article. Further inquiries can be directed to the corresponding authors.

## Abbreviations

The following abbreviations are used in this manuscript:

Preterm birth	PTB
PPROMs	Preterm premature rupture of membranes
FGR	fetal growth restriction
EFW	estimated fetal weight
CRP	C-reactive protein
